# The National Medical Union (Official)

**Published:** 1914-05-16

**Authors:** 


### THE NATIONAL MEDICAL UNION (Official).

Offices: 346 Strand. Tel.: City 8444.

### The London Organisation Committee.

A MEETING of the Organisation Committee for London was held at the offices of the Union on Wednesday, May 6. Present: Dr. Oppenheimer, in the chair; Drs. Sharman, Eccles, Greene, Millar, Handson, Marsh, Thomson Drake, Morison, and Carswell.

An interesting report was presented by Dr. Marsh, of Westminster. At the previous meeting Dr. Marsh had undertaken to take the preliminary steps in Westminster for either forming a local non-panel association or interesting medical men resident in his neighbourhood in matters concerning the Union. At that time Dr. Marsh mentioned the fact that, although he was present at the Conference at which the National Medical Union was formed, he had not until quite recently heard anything further of either the existence or activity of the Union, although he was living and practising in the immediate neighbourhood of its headquarters. This experience is interesting as indicating the necessity for energetic work on the part of the Organisation Committee.

At the meeting on the 6th instant Dr. Marsh stated that there were 671 medical men in the district of Westminster. This included 75 in Welbeck Street, 298 in Harley Street, and 80 in Queen Anne Street. According to the panel list for 1914 there were 127 of these on the panel, distributed as follows: In the Pimlico neighbourhood 23, in the wider district of Westminster 66, leaving a balance of 38 *who neither resided nor had an address in the neighbourhood.* He further reported that comparatively few replies were received by him in answer to a circular which he had issued. In this respect it was stated that Dr. Marsh's experience was similar to that of workers in other districts, where it had been found that only personal interviews had any effect in attracting the attention of the medical men to the ideals animating the Union.

During the past three weeks several members of the Organisation Committee have been busy arranging, from a Medical Directory wherein all names of panel practitioners have been deleted, alphabetical lists of non-panel practitioners for each borough or group of boroughs in London. At the meeting these lists were distributed to the secretaries or representatives of the London non-panel associations, with a request that they should be corrected for the area and returned to the central office with detailed information concerning each name on the list. In this way it is proposed to form a card index which will indicate such particulars as "non-panel," "partner on the panel," "member of local association," "member of Union," "retired," "consultant," and so on. The arranging of these alphabetical lists brought out the fact that in Westminster and Paddington there is a very large body of consultants, who, of course, are not materially affected in the same way as general practitioners. The meeting considered that these medical men should be approached with a view to securing their moral and financial support. Hitherto they have looked on at the efforts of general practitioners from an outside point of view, but there are many signs that there is no lack of interest or of willingness to take an active part in maintaining the prestige of the medical profession to which they equally belong. The situation in London in this respect compares very unfavourably with that in Edinburgh, where the rank and file of the profession has been most loyally supported by its leaders.

The meeting considered that in the meantime the duty of approaching the consultants should be performed by the local associations, and the hon. secretaries were instructed to write to the local associations to this effect and to express the opinion that steps should be taken by each association to approach a selected list of consultants with a view to obtaining their active co-operation at an early date, so that by the time of the first general meeting the profession might be represented in the Union by a considerable number of its leading members.

With reference to the collection of signatures of practitioners not on the panel but directly affected by the Insurance Act, who abstained from voting at the recent election of a Local Medical Committee for London conducted by the Metropolitan Counties Branch of the British Medical Association, the committee considered that, although the election had, thanks to the timely action of the Union, proved a complete fiasco, it would be well to complete the collection of signatures as a precautionary measure.

### MEETING OF COUNCIL.

A meeting of the Provisional Federating Council has been summoned for Saturday, May 23, at 4.30 P.M., at the offices of the Union, to receive reports of committees and to make arrangements for the general meeting. In connection with the general meeting, a report will be prepared by the Council of its actions and recommendations during its term of office. This report will be printed and circulated to members as soon as possible, so that members of the Union may be in a position to discuss it at the general meeting. It will be advisable that the discussion at the general meeting should be founded upon this report, but, of course, it will be open to any member of the Union to propose resolutions on any subject which he considers pertains to the business in hand. Members of associations are requested to bear in mind that all resolutions should be sent in to the office prior to the date of issue of the agenda for the meeting. Further notice to this effect will be given at a later date.

Non-Panel Directory.

In connection with the correspondence in The Hospital on the subject of a Directory of Medical Men not on the panel, it may interest members to know that the hon. secretaries approached the Editor of Churchill's Directory with a request that the latter should insert the words "Member of National Medical Union" in the next issue of their Directory. The Editor declined to fall in with the suggestion on the ground that at present only associations or bodies devoted to scientific subjects came within the scope of their publication. It is not improbable, however, that a resolution from the general meeting or from a considerable body of practitioners to the same effect would have greater weight with the Editor. One prominent member of the Union who some time ago made a similar application on his own account, when met with a refusal, promptly requested the omission of his name in the next issue of the Directory; this request was promptly granted.

Press Committee.

Owing to the large amount of miscellaneous work which has fallen upon the Provisional Council and its committees, no satisfactory arrangements have hitherto been made for the collection and editing of National Medical Union news, which at present appears piecemeal in the weekly issues of The Hospital. It is hoped that presently some satisfactory arrangement will be made for covering this work. In the meantime the secretaries will be most grateful for any assistance which any member feels disposed to afford either in the way of contributing articles or furnishing news which is likely to prove of general interest to the members of the Union.

